# Bead Number Effect in a Magnetic-Beads-Based Digital Microfluidic Immunoassay

**DOI:** 10.3390/bios12050340

**Published:** 2022-05-16

**Authors:** Wensyang Hsu, Yu-Teng Shih, Meng-Shiue Lee, Hong-Yuan Huang, Wan-Ning Wu

**Affiliations:** 1 Department of Mechanical Engineering, National Yang Ming Chiao Tung University, Hsinchu 30010, Taiwan; mo561213.me08.en08@nycu.edu.tw (Y.-T.S.); mengshiueleenycu@nycu.edu.tw (M.-S.L.); 2 Institute of Pharmacology, National Yang Ming Chiao Tung University, Taipei 11221, Taiwan; 3 Department of Obstetrics and Gynecology, Chang Gung Memorial Hospital, Taoyuan 33305, Taiwan; hongyuan@cgmh.org.tw (H.-Y.H.); wlw1830@cgmh.org.tw (W.-N.W.); 4 Department of Obstetrics and Gynecology, College of Medicine, Chang Gung University, Taoyuan 33302, Taiwan

**Keywords:** magnetic beads, droplet-based microfluidics, immunoassay, coefficient of variation

## Abstract

In a biomedical diagnosis with a limited sample volume and low concentration, droplet-based microfluidics, also called digital microfluidics, becomes a very attractive approach. Previously, our group developed a magnetic-beads-based digital microfluidic immunoassay with a bead number of around 100, requiring less than 1 μL of sample volume to achieve a pg/mL level limit of detection (LOD). However, the bead number in each measurement was not the same, causing an unstable coefficient of variation (CV) in the calibration curve. Here, we investigated whether a fixed number of beads in this bead-based digital microfluidic immunoassay could provide more stable results. First, the bead screening chips were developed to extract exactly 100, 49, and 25 magnetic beads with diameters of less than 6 μm. Then, four calibration curves were established. One calibration curve was constructed by using varying bead numbers (50–160) in the process. The other three calibration curves used a fixed number of beads, (100, 49, and 25). The results indicated that the CVs for a fixed number of beads were evidently smaller than the CVs for varying bead numbers, especially in the range of 1 pg/mL to 100 pg/mL, where the CVs for 100 beads were less than 10%. Furthermore, the calculated LOD, based on the composite calibration curves, could be reduced by three orders, from 3.0 pg/mL (for the unfixed bead number) to 0.0287 pg/mL (for 100 beads). However, when the bead numbers were too high (more than 500) or too low (25 or fewer), the bead manipulation for aggregation became more difficult in the magnetic-beads-based digital microfluidic immunoassay chip.

## 1. Introduction

The immunoassay chip integrates various immunodetection steps in a tiny device. Among the integration techniques, the microfluidics-based immunoassay chip is a popular device because of the small liquid volumes, rapid turnaround time, and high portability offered by microfluidics [1,2,3,4,5,6,7]. Using beads as carriers, the surface area available for antigen-antibody binding is greatly increased, thereby enhancing the signal and shortening the reaction time [8,9,10,11,12,13,14,15]. For example, in 2015, Ghodbane et al. used a bead-based microfluidic chip to measure the concentration of multiple groups of human IL-1β in a channel-based microfluidic chip [8]. The required sample volume was 4.2 μL, the detection time was 5.5 h, and the detection limit was 21.8 pg/mL. The number of beads was controlled by specifying the total bead weight; the number used was more than 1000. When there were thousands of beads with a specified total weight, the deviation in bead number in each measurement represented just a small portion of the total beads. Then, the impact on the signal variations due to this small bead number deviation was also small.

In comparison to channel-based microfluidics, droplet-based microfluidics, also known as digital microfluidics (DMF) to indicate the “digitalization” of the liquids into droplets, is outstanding in terms of a small sample volume requirement [16,17,18,19,20,21,22,23,24,25]. Therefore, the DMF-based immunoassay becomes the most attractive approach for diagnostics with a limited sample volume and low concentration. Previously, based on electrowetting-on-dielectric (EWOD) technology [26,27,28,29,30,31,32], our group reported a magnetic-beads-based digital microfluidic immunoassay with a low bead number [33,34]. By aggregating magnetic beads in the detection stage, the fluorescence intensity can be effectively enhanced to reduce the limit of detection (LOD) [33]. With a constant amount of antigens, the use of fewer magnetic beads in an immunoassay can capture more antigens on each bead. However, since the total bead number is low, a small variation in the bead number, such as an unfixed bead number in each measurement or the loss of some beads during the process, may represent a significant portion of the total beads. This could result in a large variation on the final signals. Therefore, using fewer magnetic beads in an immunoassay, without bead loss during the process, could increase average fluorescent intensity amplification per bead, which would help to further reduce the LOD to a pg/mL level with a total process time of 30 min [34]. Recently, with the above magnetic-beads-based digital microfluidic immunoassay platform, growth factors, IL-1β, and TNF-α were first detected at the pg/mL level from a human single-embryo culture medium droplet of 0.52 μL [35], where only a 5–10 μL culture medium was available. However, the number of magnetic beads used in this platform (±100) was not fixed in each measurement, which might have caused an unstable CV (coefficient of variation) in the calibration curve.

To our knowledge, flow cytometry [36] is currently the only immunoassay technique that uses a fixed number of beads, but the sample volume required by flow cytometry is much higher than that of a magnetic-beads-based digital microfluidic immunoassay, and flow cytometry cannot meet the requirements when the sample concentration is low and the available volume is small. This study aims to investigate whether a fixed number of magnetic beads in our magnetic-beads-based digital microfluidic immunoassay platform can provide more stable experimental results. First, bead screening chips were developed to extract exactly 100, 49, and 25 magnetic beads. Then, four calibration curves were established with the magnetic-beads-based digital microfluidic immunoassay. One calibration curve was constructed by using various bead numbers (50–160) in the process. The other three calibration curves used a fixed number of beads (100, 49, and 25). The measurement results were then compared.

## 2. Methods and Materials

**Figure 1:**
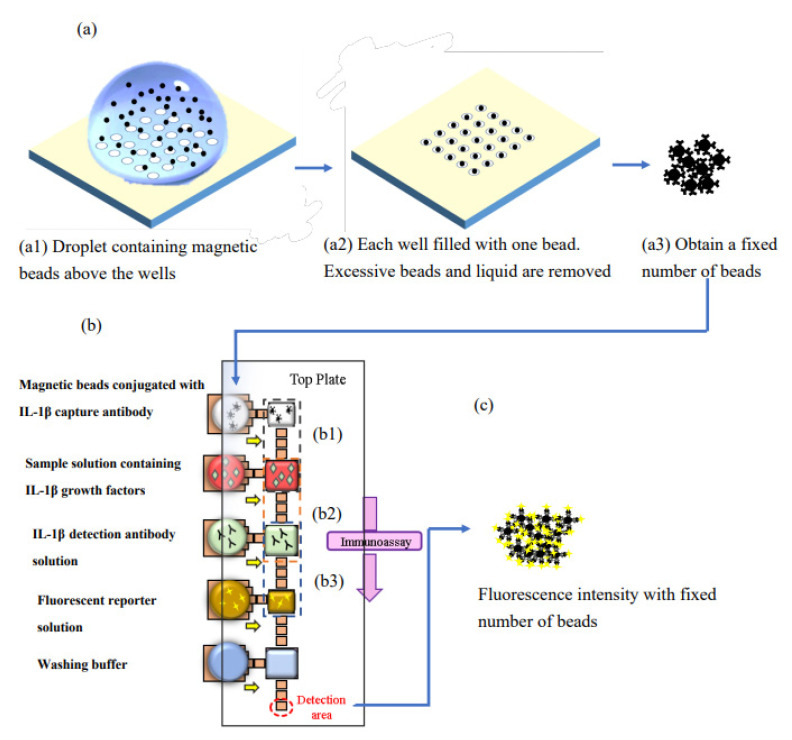
Operating concept: (**a**) obtaining the specified magnetic bead number on a bead screening chip; (**b**) immunoassay process on the chip with a fixed number of magnetic beads; (**c**) beads’ aggregation for fluorescence intensity measurement at the detection area.

Figure 1 shows the process flow of an immunoassay analysis of a digital microfluidic immunoassay chip with magnetic beads. In order to establish the calibration curve that linked IL1-β concentration and fluorescence intensity, a fixed number of magnetic beads conjugated with IL1-β captured antibody was first obtained by a bead screening chip, as illustrated in Figure 1a. Then, the magnetic beads along with other reagents were loaded into the digital microfluidic chip reservoirs to perform the immunoassay process, as shown in Figure 1b. In the end, as Figure 1c illustrates, the magnetic beads were aggregated in the detection area for fluorescence intensity measurement. The processes were repeated three times at each concentration using the same number of beads, thereby establishing the calibration curve data points for a given number of beads. The entire process was then repeated to obtain the calibration curves for different numbers of beads. 

The extra time needed in this magnetic-beads-based DMF immunoassay with a fixed number of beads is the time required to prepare the specified bead number, in advance and in batches. The total process time for the current DMF immunoassay chip is identical to that of the previous DMF immunoassay with an unfixed number of beads. Figure 1 provides the detailed descriptions of the designs and operations of the bead-screening chip and the magnetic-beads-based digital microfluidic immunoassay.

### 2.1. Bead Screening Chip

The magnetic bead-screening chip allowed the magnetic beads in the beads droplet to fall into a fixed number of wells on the chip. An external magnet was used to move and attract the magnetic beads into falling into the wells. Afterwards, excessive magnetic beads were removed to achieve the required quantitative effect. The measured size of the magnetic beads was between 4.5 μm to 5.5 μm. In order to allow only one magnetic bead to fall into one well, the size of the circular hole of the well structure was designed with a diameter of 6 μm, a depth of 8 μm, and hole-to-hole spacing of 15 μm. 

The bead screening chip was made of PDMS by micro-molding technology. The micro-mold was fabricated by the photolithography process on a silicon wafer. The negative photoresist SU-8 3005 was used to make an 8 μm thickness pattern of micro-mold. Then, the PDMS casting was performed by mixing a PDMS base with a curing reagent, with a 10:1 ratio. After curing at 80 °C for 2 h, the demolding was implemented to complete the bead-screening chip.

**Figure 2:**
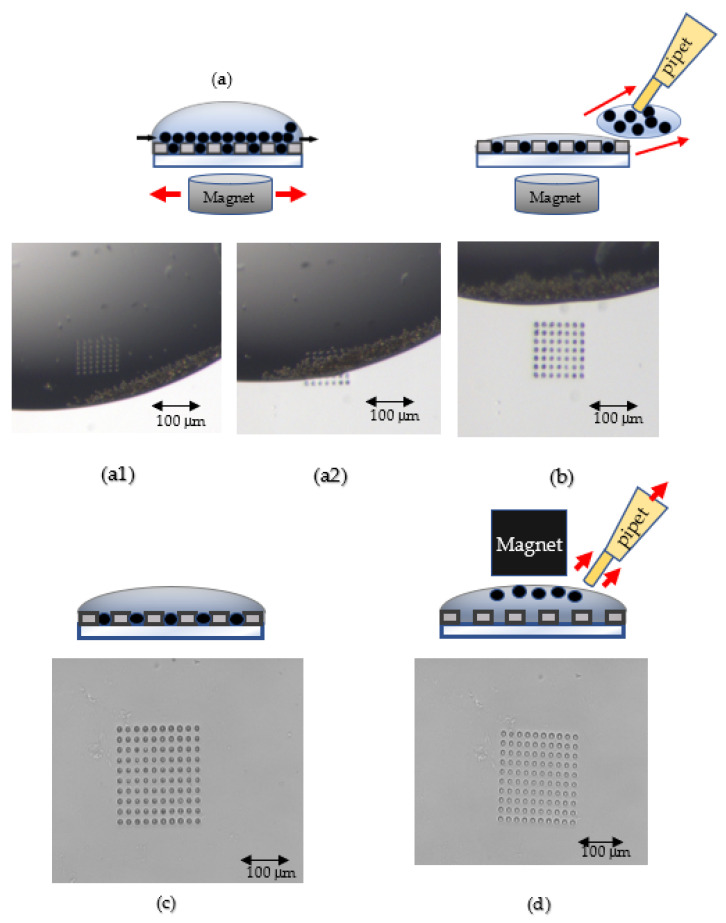
Obtaining the specified bead number on the bead screening chip. (**a**) Move the bead droplet and magnet to fill the wells with beads: (**a1**) Drag the bead droplet towards the wells; (**a2**) Move the droplet and magnet backward and forward to fill the wells with beads. (**b**) Remove excessive beads with liquid by a pipet. (**c**) Every well was filled with exact one bead; (**d**) Use a magnet to attract the beads to the droplet top surface and then retrieve the droplet with all beads by a pipet.

For the capture and removal of the magnetic beads, a beads droplet was first placed onto the screening chip by a pipet. Then, the pipet tip was used to drag the beads droplet backward and forward in the wells with a magnet under the chip until the wells were completely filled with the magnetic beads, as shown in Figure 2a. Finally, the excessive magnetic beads with liquid were pulled away by the pipet tip, as shown in Figure 2b,c. To remove the magnetic beads in the wells, a PL + PBS droplet (Pluronic 127/PBS = 6 mg/mL) was dropped onto the screening chip, and then a magnet was used to attract all the magnetic beads in the wells toward the top of the droplet, as shown in Figure 2d. As long as the external magnet did not touch the droplet, the surface tension could hold the magnetic beads in the droplet. The droplet and all the magnetic beads were then retrieved by the pipet. 

### 2.2. Magnetic-Beads-Based Digital Microfluidic Immunoassay

Figure 1b shows the layout of the immunoassay chip. On the left side, there are five reservoirs for loading the beads solution, the sample solution, the detection antibody solution, the fluorescent reporter solution, and the washing buffer. On the right side, there are five larger major electrodes for the droplets generated from the corresponding reservoirs. Between those major electrodes, there are smaller electrodes used as a transporting and mixing area, a washing area, and a detection area. 

The function of the transporting and mixing area was carrying out the reagent’s transportation, mixing, and incubation; the washing area provided for the removal of residual reagents of the magnetic beads after the immunoreaction; the detection area was where the beads were aggregated for fluorescent detection after the immunoreaction. The immunoreaction consisted of five steps in sequence. Figure 1(b1) shows the mixing of the fixed number of magnetic beads droplet sand the sample droplets (human IL-1β antigen) for 12 min. Then, a smaller daughter droplet containing all beads was extracted and moved to the next electrode to mix with the detection antibody for 6 min, as shown in Figure 1(b2). Figure 1(b3) shows that the beads droplet was mixed with the fluorescent reporter droplet for 6 min. Afterwards, the washing step was carried out and then the mix was moved to the lower detection area for fluorescent intensity measurement, as shown in Figure 1c.

In a parallel-plate EWOD chip, when the gap between the top plate and the bottom plate is constant, the droplet volume is proportional to the area of the electrode. In the standard ELISA kit, the sample solution and the detection antibody solution are both 80 µL, and the fluorescent reporter solution is 50 µL. By keeping the same volume ratio, 8:5, the electrodes for handling the sample droplet and the detection antibody droplet were both 2828 × 2828 μm^2^; the electrodes for the beads droplet and the fluorescent reporter droplet were both 2236 × 2236 μm^2^. In order to have good washing efficiency without bead loss in the droplet cutting and washing steps, the electrode size for the transportation and mixing area was 707 × 707 μm^2^, with a minimum volume ratio of 5:1 [20]. For better droplet maneuverability, the gap between the top and the bottom plate was 65 µm in all processes, except for the detection stage, where the gap was reduced to 10 µm to avoid the vertical stacking of beads. 

**Figure 3:**
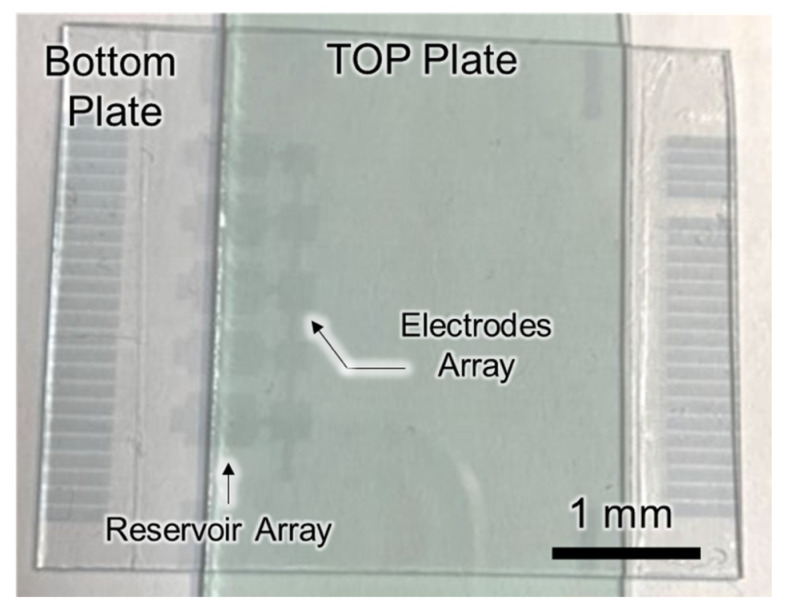
The digital microfluidic chip for immunoassay.

The magnetic-beads-based digital microfluidic chip was made by assembling the top plate and the bottom plate, as shown in Figure 3. The fabrication processes of the bottom plate included ITO patterning, SU-8 deposition, Al_2_O_3_ deposition, and CYTOP deposition. The ITO layer was patterned as the electrode; the SU-8 and the Al_2_O_3_ layer were the dielectric layers; and the CYTOP was the hydrophobic layer. The ITO patterning was performed through lithography and ITO etching. The etching solution was aqua regia (NHO_3_:HCl:H_2_O_2_ = 1:3:6). The 2.8 μm SU-8 was deposited without patterning. The 50 nm Al_2_O_3_ deposition was carried out through the in-house 8-inch ALD system developed by the Taiwan Instrument Research Institute with precursors Trimethyl aluminum (TMA) and H_2_O at process temperature 200 °C. The 700 nm CYTOP deposition was processed through spin-coating and baking. Then, the top plate was fabricated through the CYTOP deposition on the pattern-free ITO glass. Finally, by assembling the top plate and the bottom plate, the magnetic-beads-based digital microfluidic chip was completed. The gap between the two parallel plates was controlled by a spacer with a thickness of 65 μm, except at the detection step where the gap was reduced to 10 μm to avoid the vertical stacking of beads.

Before immunoreaction proceeded, human IL-1β antigens were diluted in several sample solutions with different concentrations of 0.1 pg/mL, 1 pg/mL, 10 pg/mL, 25 pg/mL, 50 pg/mL, 75 pg/mL, and 100 pg/mL. A human IL-1β detection antibody solution with a concentration of 20 μg/mL was prepared with PBS + PL (Pluronic F-127/PBS = 6 mg/mL). As for the fluorescent reporter, a 2 mg/mL stock solution was prepared by using DI water, then diluting it 100 times with PL + PBS (Pluronic F-127/PBS = 6 mg/mL) to form a fluorescent reporter solution. 

The magnetic beads were COOH-modified magnetic beads (6 μm COMPEL™, Bangs Laboratories, Inc., Fishers, IN, USA). The captured antibodies, referring to the human IL-1β antibody, came from R&D Systems, Minneapolis, MI, USA. The conjugation between the captured antibody and the magnetic bead was performed by MagQu Co., Ltd., New Taipei, Taiwan, according to a modified protocol [37]. Briefly, each bead set (10 μL 1.25 × 10^6^ microspheres) was magnetically applied to remove the supernatant. Beads were washed twice with activation buffer, and resuspended with an activation mix (100 μL) containing N-hydroxysulfosuccinimide sodium salt (10 mg/mL, Sulfo-NHS; Thermo Scientific, Waltham, MA, USA) and 1-ethyl-3-(3-dimethylaminopropyl) carbodiimide hydrochloride (10 mg/mL, EDC; Thermo Scientific) in an activation buffer. The reaction mixture was incubated for 20 min near 23 °C in darkness in an end-over-end rotator, followed on washing twice with a coupling buffer (500 μL). The activated beads were then incubated with an antibody solution (80 μL with a final concentration 40 μg/mL in a coupling buffer) for at least 2 h near 23 °C in darkness in an end-over-end rotator. The beads were eventually washed twice with a washing buffer (500 μL) and resuspended in PBS (100 μL containing bovine serum albumin 1% *w/v*, and NaN_3_, 0.05% *w/v*). Coated beads were stored in darkness at 2 °C. The antigens used to establish the calibration curves were the recombinant human IL-1β from R&D Systems, USA. The fluorescent reporter was R-PE Streptavidin, from R&D Systems, USA. The absorption and emission wavelengths of the fluorescence fell in the range of 488–494 nm and 515–545 nm, respectively. The Pluronic F-127 and the BSA also came from R&D Systems, USA.

## 3. Results and Discussions

**Figure 4:**
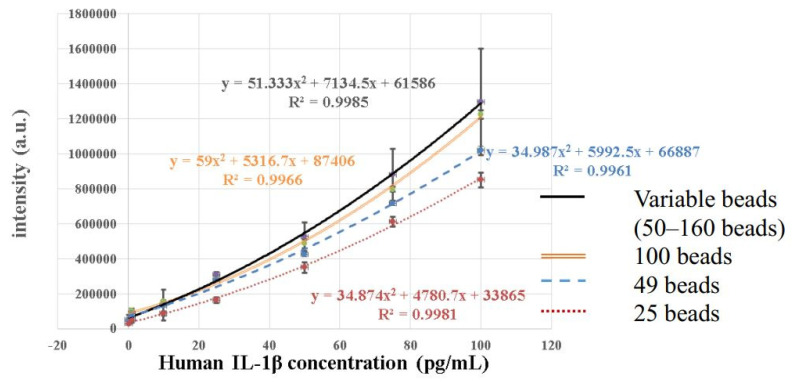
Comparison of the calibration curves obtained by an unfixed number of magnetic beads (50–160) and three fixed numbers of magnetic beads (100, 49, and 25).

**Figure 5:**
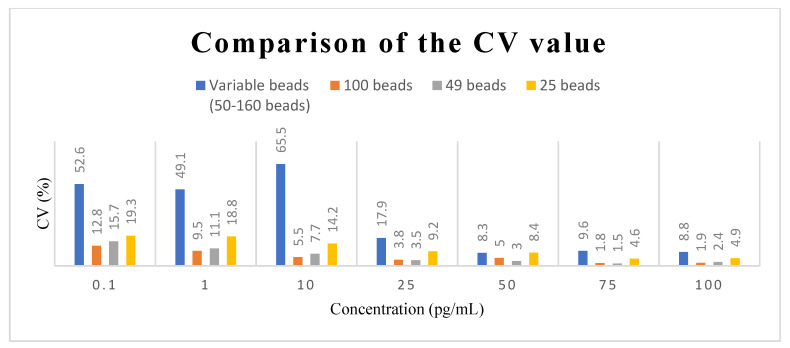
Comparison of the CV values of an unfixed number of magnetic beads and three different fixed numbers of magnetic beads at different concentrations.

In establishing the calibration curve for human IL-1β, the known concentrations of human IL-1β samples were 0 pg/mL, 0.1 pg/mL, 1 pg/mL, 10 pg/mL, 25 pg/mL, 50 pg/mL mL, 75 pg/mL, and 100 pg/mL. There were four calibration curves, including the curve for an unfixed number of magnetic beads (50–160) and the curves for fixed numbers of magnetic beads (100, 49, and 25, respectively). In all four calibration curves, the experiments were repeated three times at each concentration for standard deviation and mean value calculations. Figure 4 shows the comparison of these four calibration curves. It was found that the fluorescence intensity became stronger with the increasing concentrations in all calibration curves, and the R^2^ values were all above 0.99, which meant that all four calibration curves fit the data well. However, the standard deviation of the unquantified magnetic beads was much larger, and the intensity distribution of the different concentrations even overlapped. The standard deviations of the calibration curves developed by the other three fixed numbers of magnetic beads were significantly smaller, and the fluorescence intensity distribution of different concentrations did not overlap. Figure 5 provides a comparison of the CV values of the fixed and unfixed numbers of magnetic beads at each concentration. Figure 5 indicates that with the fixed number of magnetic beads at the concentration range of 1.0 pg/mL~100 pg/mL, the CV values for 100 and 49 beads were all lower than 10%, except for the curve for 49 beads at 1.0 pg/mL, which was slightly larger than 10%. Compared to the values of the unquantified beads, the CV values for the fixed number of beads were much lower. The CV values increased when the concentration became smaller, because the total fluorescence intensity also became smaller at low concentrations. In fact, the standard deviation was not necessarily larger. When it came to 25 beads, although the standard deviation and the CV values were larger because the magnetic beads were sometimes difficult to aggregate, they were still better than those of the unquantified beads.

**Figure 6:**
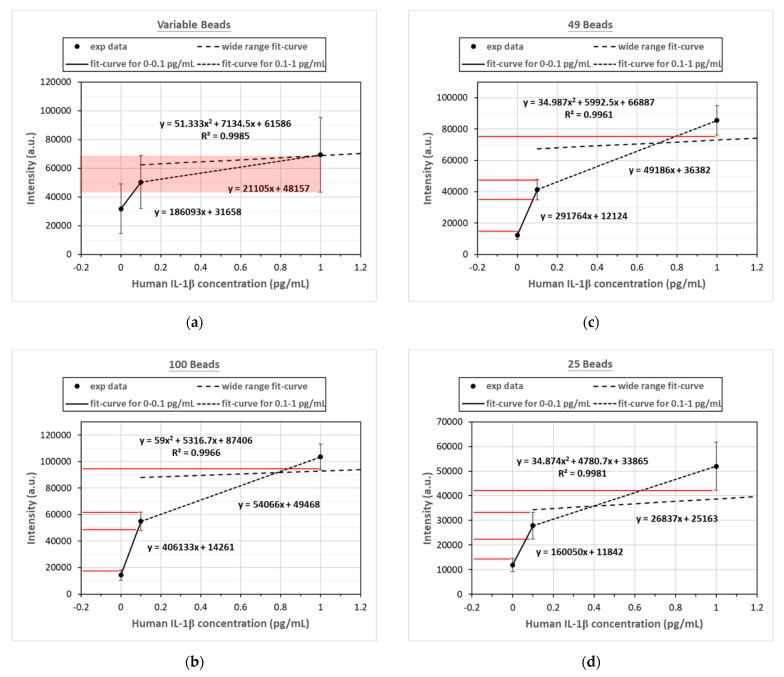
Fluorescence intensity distribution and composite calibration curves for the unfixed number of magnetic beads and for the other three with fixed numbers of magnetic beads at the low concentrations of 0.0 pg/mL, 0.1 pg/mL, and 1.0 pg/mL. (**a**) The measured fluorescence intensity distribution overlaps when the number of magnetic beads is unfixed (50–160). When the fixed numbers of magnetic beads are (**b**) 100 beads, (**c**) 49 beads, or (**d**) 25 beads at concentrations of 0.0 pg/mL, 0.1 pg/mL, and 1.0 pg/mL, respectively, the fluorescence intensity distributions do not overlap. The LODs for (**a**) variable beads, (**b**) 100 beads, (**c**) 49 beads, and (**d**) 25 beads are 3.0 pg/mL, 0.0287 pg/mL, 0.0255 pg/mL, and 0.0508 pg/mL, respectively. n = 3.

Figure 6 shows the difference in measured fluorescence intensity at the low concentrations of 0.0 pg/mL, 0.1 pg/mL, and 1.0 pg/mL between the fixed and unfixed number of magnetic beads. When the number of magnetic beads was unfixed, as shown in Figure 6a, the signals of two concentrations partially overlapped, and it was difficult to accurately differentiate 1.0 pg/mL from 0.1 pg/mL. Figure 6b–d show obvious differences when the bead number was fixed at 100, 49, or 25. The measured fluorescent signals did not overlap, and could clearly distinguish 0.0 pg/mL, 0.1 pg/mL, and 1.0 pg/mL. However, the wide-range calibration curves did not fit the experiment data very well at low concentrations. In order to have better fitting curves at low concentrations, composite linear curves passing through experiment data points at low concentrations were proposed. This meant that one linear equation linked experiment data at 0.0 pg/mL and 0.1 pg/mL, and another linear equation linked experiment data at 0.1 pg/mL and 1.0 pg/mL, as shown in Figure 6. In this way, these linear calibration curves for the low concentrations could exactly fit the experimental data points at 0.0 pg/mL, 0.1 pg/mL, and 1.0 pg/mL. The calculated intensities at these three concentrations were identical to the measured values.

Using the composite calibration curves, the calculated LODs for variable beads, 100 beads, 49 beads, and 25 beads were 3.0 pg/mL, 0.0287 pg/mL, 0.0255 pg/mL, and 0.0508 pg/mL, respectively. The method to calculate the LOD value was the typical blank determination method, in which the concentration was calculated by the calibration curve with the fluorescent intensity of the mean value plus three standard deviations at blank. The LOD was reduced by about three orders from 3.0 pg/mL (for varying numbers of beads) to 0.0287 pg/mL (for 100 beads).

**Figure 7:**
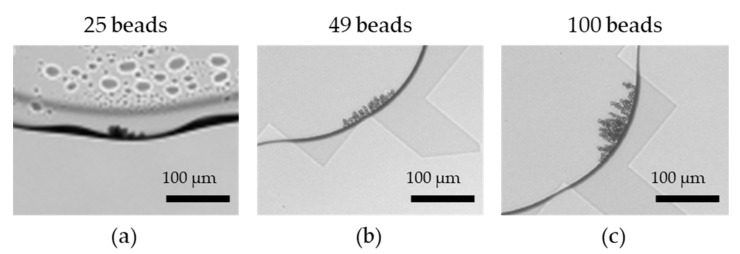
Microscopic images of successful aggregation at bead numbers of (**a**) 25, (**b**) 49, and (**c**) 100.

**Figure 8:**
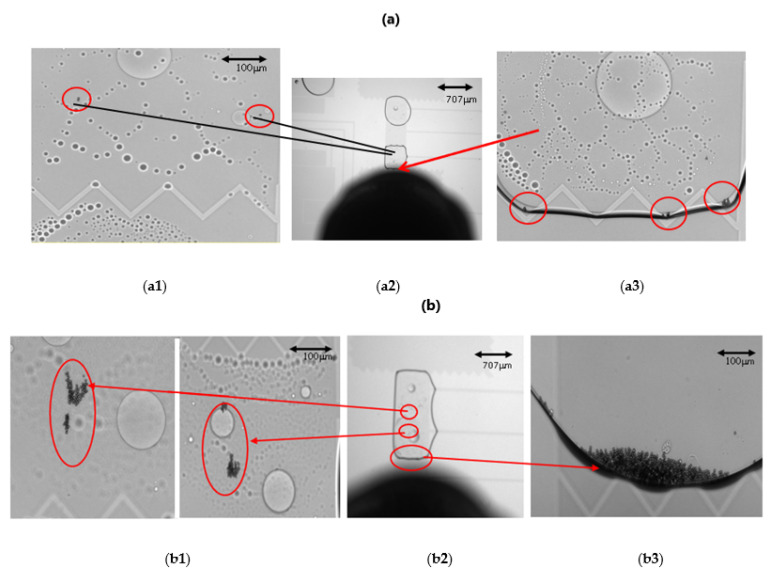
Beads aggregation issue with too few or too many beads. (**a**) 25 magnetic beads: (**a1**) Some magnetic beads cannot be aggregated; (**a2**) The droplet with 25 magnetic beads, black shadow was the magnet; (**a3**) Some magnetic beads could not be aggregated. (**b**) More than 500 magnetic beads: (**b1**) Some magnetic beads were stuck and could not be moved; (**b2**) The droplet with more than 500 beads; (**b3**) Some successfully aggregated beads.

While performing fluorescence measurement, the magnetic beads were first gathered to enhance the fluorescence intensity [21]. In order to avoid the stacking of magnetic beads, the distance between the upper and lower plates was reduced from 65 μm to 10 μm. The microscopic images of successful aggregation at bead numbers of 25, 49, and 100 are shown in Figure 7. However, when the number of magnetic beads was 25, as shown in Figure 8a, the number of magnetic beads was too small and sometimes resulted in insufficient magnetic force, which made beads aggregation more difficult. In Figure 8(a2), the droplet containing 25 magnetic beads is located above the detection electrode. The black part is the external magnet, which was used to gather the magnetic beads. Figure 8(a1,a3) shows partially enlarged views of different locations of the droplet. Some magnetic beads could not be aggregated due to insufficient magnetic force.

When the number of magnetic beads was more than 500, as shown in Figure 8b, it was easy for the beads to become stuck in the 10 μm gap between the upper and lower plates because too many magnetic beads caused immobility. Figure 8(b2) shows the droplet containing magnetic beads on the detection electrode; the black part is the external magnet. Figure 8(b1) is an enlarged view of part of the droplet, and the circled part is the magnetic beads that were stuck and could not be moved. Figure 8(b3) is an enlarged view of the bottom of the droplet, showing that some of the magnetic beads were successfully aggregated.

## 4. Conclusions

In this study, bead-screening chips were successfully developed to quantify the required magnetic beads. By fixing 100, 49, or 25 magnetic beads, the fluorescence intensity of the human IL-1β samples were measured with concentrations at 0 pg/mL, 0.1 pg/mL, 1 pg/mL, 10 pg/mL, 25 pg/mL, 50 pg/mL, 75 pg/mL, and 100 pg/mL to establish four composite calibration curves. Compared to the unfixed number of beads (50–160), with the sample size of three at each concentration in all four calibration curves, there was a significant decrease in the CVs for the fixed number of magnetic beads. Among the three different numbers of fixed magnetic beads, the CV values for 100 beads were best. In the range of 1.0 pg/mL to 100 pg/mL, the CVs for 100 beads were all less than 10%. When the number of beads was not fixed, the fluorescence intensity distribution at different concentrations may have overlapped, while there was no overlapping area with a fixed number of beads. Furthermore, the calculated LOD based on the composite calibration curves could be reduced by three orders, from 3.0 pg/mL (for the unfixed number of beads) to 0.0287 pg/mL (for 100 beads).

When there were 25 magnetic beads, the magnetic beads sometimes could not be aggregated properly due to insufficient magnetic force. When the number was more than 500, the magnetic beads easily became stuck and hard to move for complete aggregation. These results showed that using a fixed number of magnetic beads can evidently reduce the CV values and detect the concentration of the analyte to 0.1 pg/mL, which effectively improves the previous magnetic-beads-based digital microfluidic immunoassay. The use of smaller magnetic beads might reduce the chance of the beads becoming stuck in the gap, but the dispersed small magnetic beads will be harder to manipulate and aggregate by the external magnet. The optimal magnetic bead size for our DMF immunoassay needs to be further investigated in the future.

## Data Availability

Not applicable.
